# *Heterodera schachtii* enolase does not elicit canonical immune responses in *Arabidopsis thaliana*

**DOI:** 10.1038/s41598-026-56477-7

**Published:** 2026-06-08

**Authors:** Maximilian F. Euler, Neil Pep Dave N. Sumaya, Seema Aslam, Johannes Lerch, Badou Mendy, Florian M. W. Grundler

**Affiliations:** 1 INRES - Molecular Phytomedicine, Rheinische Friedrich-Wilhelms-Universität Bonn, Bonn, 53115 Germany; 2 Institut für Molekulare Physiologie und Biotechnologie der Pflanzen (IMBIO), Rheinische Friedrich-Wilhelms-Universität Bonn, Bonn, 53115 Germany; 3 Plant Pathology Division, College of Agriculture, Kabacan, 9407 Cotabato Philippines

**Keywords:** Enolase, Moonlighting, Surface protein, Plant-parasitic-nematodes, Biotechnology, Molecular biology, Plant sciences

## Abstract

Enolase (2-phospho-D-glycerate hydrolase, EC 4.2.1.11) is a conserved glycolytic enzyme that catalyzes the reversible dehydration of 2-phosphoglycerate to phosphoenolpyruvate. In animal- and entomopathogenic nematodes, enolase has been shown to interact with host organisms and activate immune responses. In the plant-parasitic cyst nematode *Heterodera schachtii*, an established model species, enolase is hypothesized to be similarly exposed to host plant tissues during infection, based on its identification in the secretomes of related plant-parasitic nematodes. Unlike animals, plants lack an adaptive immune system and instead rely on innate immune responses to detect pathogen-derived molecules. Therefore, we investigated whether *H. schachtii* enolase can activate plant immune responses and influence growth and development in its host, *Arabidopsis thaliana*. Recombinant *H. schachtii* enolase was heterologously expressed in *Escherichia coli* and confirmed to be enzymatically active. Treatment of *A. thaliana* seedlings with purified enolase did not induce reactive oxygen species production, a hallmark of early plant immune activation. A concentration of 85 $\upmu$g/ml had no effect on plant growth; only at the highest applied concentrations (425–850 $\upmu$g/ml) was shoot growth reduced, while root growth remained unaffected. Consistently, quantitative PCR analysis of canonical defense marker genes (*FRK1*, *NHL10*, *PAD3*, *CYP81F2*, and *JAZ10*) showed no robust transcriptional activation of plant immune pathways. Together, these results indicate that *H. schachtii* enolase does not function as an elicitor of canonical plant immune responses.

## Introduction

Enolase (2-phospho-D-glycerate hydrolase, EC 4.2.1.11) is a conserved metalloenzyme in the glycolysis and gluconeogenesis pathways. Enolase catalyzes the reversible dehydration of 2-phosphoglycerate to phosphoenolpyruvate^1–3^. Mammalian genomes encode three distinct isoforms with tissue-specific expression: $\alpha$-enolase is the main enolase expressed in most adult tissues, $\beta$-enolase is expressed in muscle-tissue while $\gamma$-enolase is neuron-specific^4–6^. As an essential enzyme in glycolysis, the sequence is highly conserved among species, with 40-90% shared identity^7,8^.

However, research has discovered multifaceted activities of enolase which exceed its primary glycolytic function. These include its function as a molecular chaperone^9^, interaction with microtubules^10^, the function of $\alpha$-enolase as one of several plasminogen receptors, its alternative translation product Myc promoter-binding domain MBP-1^11^ and a diverse involvement in cancer processes^8,12^. Moreover, in a bioinformatic approach enolase multifunctionality was assessed using protein-protein interaction networks in four species, namely *Homo sapiens*, *Drosophila melanogaster*, *Caenorhabditis elegans*, and *Saccharomyces cerevisiae*, revealing multiple potential connections to growth, cell death, transcription or aging^13^. The majority of recent studies, however, have focused on the function of enolase in immunity.

Knockdown of enolase in *Arabidopsis thaliana* *los2-2* mutants caused severe growth inhibition, as expected for a compromised glycolytic pathway. Additionally, it induced autoimmunity and increased salicylic acid content, revealing a link between enolase and plant immune pathways^14^. Enolase from rubber tree *Hevea brasiliensis*, was found to be a minor latex allergen^15^.

In pathogens, enolase, often found extracellularly, has been under investigation for its capability to activate the host immune system and act as antigen. Fungal enolase was identified as immunodominant allergen^16^. For example, this is the case in the human pathogenic fungus *Candida albicans*^17–20^ and in baker’s yeast (*Saccharomyces cerevisiae*^21^), where enolase is found in the cell wall^22^ and is involved in vacuole fusion^23^. In *C. albicans*, enolase was suggested to be involved in evading the host immune system^19^.

Enolase has been identified in excretory/secretory products of helminths, such as trematode *Echinostoma caproni*^24^, nematodes *Meloidogyne incognita*^25^ and *Ascaris suum*^26^ and commonly in extracellular vesicles (EVs)^27–29^. In the entomopathogenic nematode *Steinernema glaseri*, enolase was identified as a surface coat protein and detectable in the hemolymph of the *G. mellonella* larva host after infection^30^. In animal-parasitic nematodes *Trichinella spiralis and Ascaris suum*, the potential to use enolase as a vaccine was studied in mice^31,32^. Recombinant enolase protein and a plasmid encoding *T. spiralis* enolase induced cell-mediated (Th1) as well as humoral (Th2) immune responses. Enolase from *Anisakis simplex*, a parasite of fish, reacted with mouse sera but not with human sera, possibly due to insufficient length of exposure to enolase^33^.

Plant-parasitic nematodes (PPNs), particularly cyst and root-knot nematodes, cause substantial yield losses in major crops, reaching up to 9% in soybean and highly affecting other crops such as banana, vegetables, cereals and potato^34^. Enolase from the root-knot nematode *M. incognita* has been identified extracellularly as part of the nematode secretome^25^, suggesting that this conserved metabolic enzyme may be exposed to host plant tissues. While PPN research has largely focused on secreted effectors that manipulate host cellular processes, comparatively little is known about extracellular nematode proteins that are not classical effectors and their potential interactions with the host prior to and during infection. Enolase has been implicated in host–pathogen interactions and immune modulation in various animal and insect pathosystems, raising the possibility that extracellular nematode enolase may also interact with plant host systems. Therefore, in this study, we cloned and heterologously expressed enolase from the cyst nematode *Heterodera schachtii* and investigated its ability to activate plant immune responses and influence growth and development in *A. thaliana*.

## Results

### Bioinformatic characterization of *H. schachtii* enolase

**Fig. 1:**
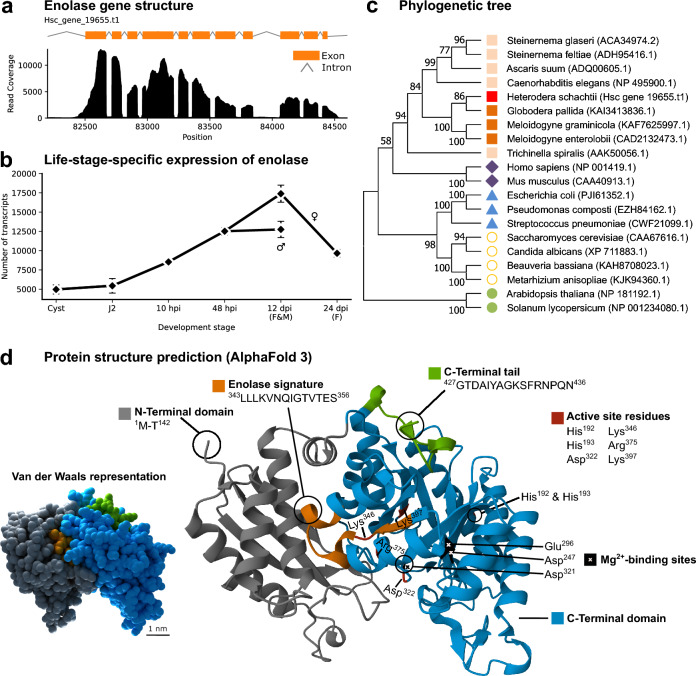
Characterization of *H. schachtii* enolase. **(a)** Hsc_gene_19655.t1 is the only gene coding for an enolase in *H. schachtii*. The genomic coding sequence is organized into 11 exons, read coverage is shown at the bottom^35^. **(b)** Life-stage specific expression shows increasing enolase transcripts during nematode development until 12 days post-infection (dpi), with maximum expression in females (ppJ2: pre-parasitic J2s, hpi: hours post-infection, dpi: days post-infection). **(c)** Phylogenetic tree of enolase sequences from 20 organisms from five species. Phylogenetic tree for evolutionary analyses was produced using the Neighbor-joining method. Bootstrap values indicated were inferred from 1000 replicates. **(d)** Conserved features of *H. schachtii* enolase highlighted on an AlphaFold 3 structure prediction^37^ (Predicted template modeling score = 0.97), based on yeast enolase.

For identification of enolases in *H. schachtii*, enolase sequences from other nematode species were searched against the *H. schachtii* genome^35^ using NCBI BLAST. Despite the presence of multiple enolases in other organisms, only a single gene was identified in *H. schachtii* (Hsc_gene_19655.t1). The HsENO genomic sequence consists of 11 exons and 10 introns^35^ (Fig. 1a). The 1311 bp cDNA encodes a protein with 436 amino acid residues and a predicted molecular weight of $\approx$ 47.68 kDa. Based on life-stage-specific transcriptomic analysis^35^, the highest expression of HsENO was observed in females at 12 days post-infection (dpi, Fig. 1b). Females and males of *H. schachtii* differ markedly in size and metabolic activity, with females developing into sedentary, syncytium-maintaining feeders that represent the standard reference point for phenotypic assessment of infection^36^. In males, expression at 12 dpi is lower than in females. Phylogenetic analysis (Fig. 1c) highlights the conserved nature of enolase sequences across species: sequences from 20 organisms spanning plants, fungi, bacteria, vertebrates (human and mouse), and both plant- and animal-parasitic nematodes, clustered into three distinct clades. Firstly, entomopathogenic, animal-parasitic and plant-parasitic nematodes and mammals clustered together with 58% probability, with strong conservation among nematodes (*P* = 94%). Secondly, bacteria and fungi (*P* = 98%) and thirdly, plants. Sequence identity of enolase, compared to *H. schachtii*, ranges from 47.42% (*Streptococcus pneumoniae*), 64.12% in baker’s yeast (*Saccharomyces cerevisiae*), 68.52% in (*A. thaliana*), 71.36% in animal-parasitic nematode (*Trichinella spiralis*), 77.65% in entomopathogenic nematode (*Steinernema glaseri*) to 84.03% in plant-parasitic nematode (*Meloidogyne graminicola*). *H. schachtii* enolase, as a highly conserved enzyme, also contains the characteristic and essential features of enolases, as highlighted in the AlphaFold 3 tertiary structure prediction^37^ (Fig. 1d): Compared to yeast enolase^38^ all residues are shifted forward by one amino acid. *H. schachtii* enolase contains all three Mg$^{2+}$ binding sites at Asp$^{247}$, Glu$^{296}$ and Asp$^{321}$ and all six active site residues, with only one amino acid substitution of Asn$^{193}$ to His$^{193}$. The amino acids His$^{192}$, Asp$^{322}$, Lys$^{346}$, Arg$^{375}$ and Lys$^{397}$ remain conserved. Despite the presence of enolase in secretomes of related nematodes, the sequence does not code for a signal peptide for canonical secretion, as predicted by SignalP-6.0^39^.

### Cloning, expression and purification of recombinant *H. schachtii* enolase

**Fig. 2:**
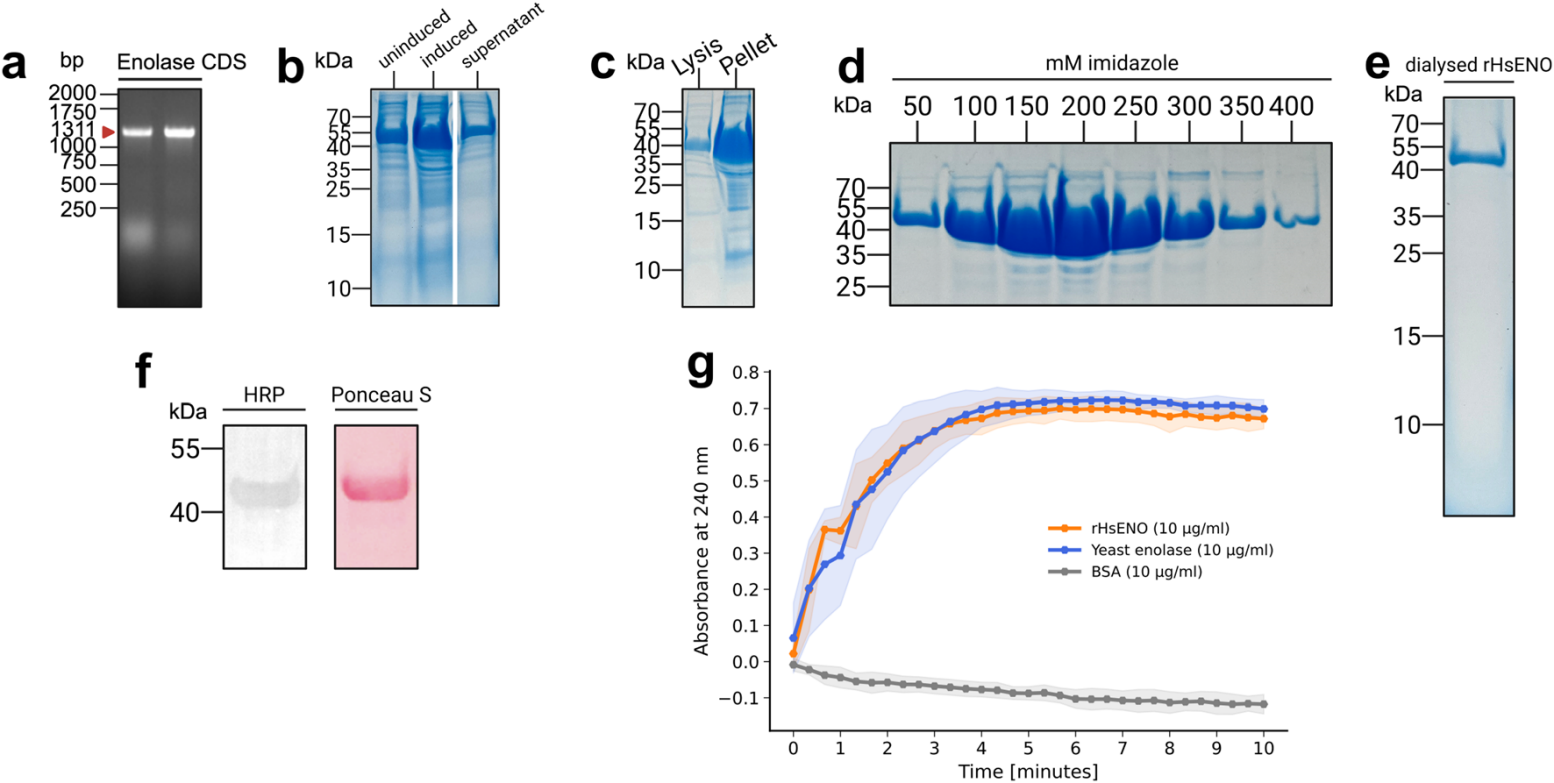
Cloning, expression, purification, and enzymatic activity of recombinant enolase from *H. schachtii*. **(a)** PCR amplification of the Hs-enolase coding sequence (1311 bp) from cDNA synthesized from J2-stage total RNA. **(b)** SDS-PAGE analysis of *E. coli* cells before and after induction with 1 mM IPTG showing expression of recombinant enolase around 47.7 kDa. **(c)** Extraction of rHsENO from *E. coli* resulted in partial recovery in the soluble lysate and predominant localization of the protein in the pellet fraction. **(d)** Stepwise purification by Ni-NTA affinity chromatography using increasing concentrations of imidazole. **(e)** Final purified and dialyzed rHsENO showing absence of protein contaminants. **(f)** Western blot analysis using anti–6xHis antibody confirming expression of recombinant enolase. **(g)** Functional validation of rHsENO activity through conversion of 2-phosphoglycerate to phosphoenolpyruvate, monitored by absorbance increase at 240 nm; baker’s yeast enolase served as a positive control and BSA as a negative control. Shown is the average of four independent biological replicates ± SD. Uncropped/full-length gel and blot images are provided in Supplementary Figs. S4–S6.

For amplification of enolase, *H. schachtii* J2s were collected, crushed in liquid nitrogen, and total RNA was extracted. Following synthesis of complementary DNA, the enolase CDS was amplified by PCR, yielding a single product of approximately 1311 bp (Fig. 2a). Sequencing of the enolase insert confirmed that the gene in *H. schachtii* is encoded by a 1311 bp cDNA sequence. Following Gateway cloning, SDS-PAGE analysis of *E. coli* Rosetta (DE3)pLysS cells before and after IPTG induction confirmed expression of a protein with the predicted molecular weight of approximately 47.68 kDa (Fig. 2b). Comparison of uninduced and induced fractions indicated that basal expression had already occurred prior to IPTG induction (Fig. 2b). Recombinant enolase was also detected in the culture supernatant, likely due to partial cell lysis or membrane leakage during overexpression. Following cell lysis and centrifugation, recombinant enolase was recovered in both soluble and insoluble fractions, although the majority remained in the insoluble pellet fraction (Fig. 2c). Recombinant enolase was purified from the crude lysate by immobilized metal affinity chromatography (IMAC) using Ni-NTA agarose and eluted with increasing concentrations of imidazole (Fig. 2d). Purified rHsENO was dialyzed against 1 $\times$ PBS or ddH$_2$O using dialysis cassettes with a molecular weight cutoff of 20 kDa. The resulting rHsENO showed no visible protein contamination (Fig. 2e) and was used for further experiments. Western blot analysis using an anti–6 $\times$ His antibody confirmed the identity of the purified recombinant enolase (Fig. 2f). Enzymatic activity was verified by measuring the conversion of 2-phosphoglycerate to phosphoenolpyruvate (Fig. 2g). Absorbance at 240 nm increased over time in reactions containing rHsENO and yeast enolase (positive control) but not BSA (negative control), confirming that the recombinant protein is enzymatically active.

### Evaluation of the effect of recombinant *H. schachtii* enolase protein on plant immunity

#### Production of reactive oxygen species in response to nematode enolase

**Fig. 3:**
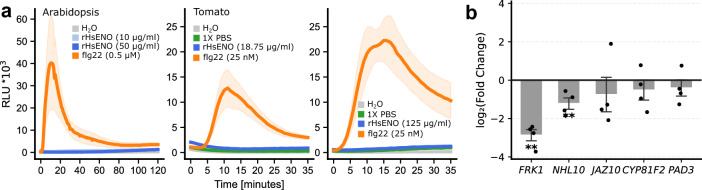
Evaluation of the effect of recombinant *H. schachtii* enolase protein on plant immunity. **(a)** Measurement of ROS production in Arabidopsis and Tomato leaf discs. The second and third panels show independent tomato experiments performed under identical conditions, differing only in the tested rHsENO concentration. At 125 $\mu$g/ml and 18.75 $\mu$g/ml rHsENO did not induce apoplastic H$_{2}$O$_{2}$ production in tomato (*Solanum lycopersicum*), nor at 10 $\mu$g/ml or 50 $\mu$g/ml in *A. thaliana* leaf discs. Data represent the mean ± SE of four (tomato) or six (Arabidopsis) leaf discs; three independent experiments yielded similar results (see Supplementary Figs. S1–S3). **(b)** Expression of canonical defense marker genes in Arabidopsis roots after 6 hour incubation with 50 $\mu$g/ml rHsENO. Values represent the mean ± SE of four biological replicates (black dots).

Production of apoplastic reactive oxygen species is one of the first reactions of a plant when a PAMP such as the conserved epitope of bacterial flagellin (flg22) is perceived by a plant receptor^40^. To determine whether rHsENO activates early immune responses, we measured apoplastic ROS production in Arabidopsis and tomato (*Solanum lycopersicum*). Leaf discs of tomato and *A. thaliana* were treated with rHsENO and analyzed in a chemiluminescence assay. Recombinant enolase did not induce a typical receptor-dependent ROS burst, as observed with the flg22 control, in either Arabidopsis or tomato, suggesting that rHsENO does not activate receptor-mediated ROS production under the tested conditions (Fig. 3a).

#### qPCR analysis of defense marker genes

To assess activation of major branches of the *A. thaliana* immune system, we monitored transcript levels of established defense marker genes representing complementary pathways following 6 h incubation with 50 $\mu$g/ml rHsENO. *FRK1* indicates pattern-triggered immunity (PTI) downstream of the FLS2–MAP kinase cascade^41^. *NHL10* marks hypersensitive response (HR) and senescence-associated defense linked to localized cell death^42^. *PAD3* encodes a cytochrome P450 essential for camalexin biosynthesis, a phytoalexin conferring resistance to necrotrophic fungi^43^. *CYP81F2* participates in indole glucosinolate modification, producing bioactive metabolites that deter herbivory^44^. In addition, *JAZ10*, a member of the JASMONATE ZIM-DOMAIN (*JAZ*) family, was included as a marker for jasmonate-mediated defense signaling. *JAZ10* is a primary jasmonate-responsive gene that is rapidly induced by mechanical wounding and herbivory in a *COI1*-dependent manner and is widely used as a transcriptional readout of jasmonate pathway activation^45^. Together, these genes capture PTI signaling, HR-associated defense, and jasmonate-regulated secondary-metabolite pathways. *FRK1* and *NHL10* showed significantly reduced expression in treated plants (log$_2$FC = −2.87, p = 0.0023 and log$_2$FC = −1.22, p = 0.0057, respectively), consistent across the four biological replicates. *JAZ10*, *PAD3*, and *CYP81F2* showed no statistically significant changes (p = 0.454, 0.305, 0.409, respectively; Fig. 3b). Collectively, these results indicate that rHsENO does not induce transcriptional activation of canonical defense marker genes under the conditions tested.

#### Effect of recombinant enolase on *A. thaliana* growth and development

**Fig. 4:**
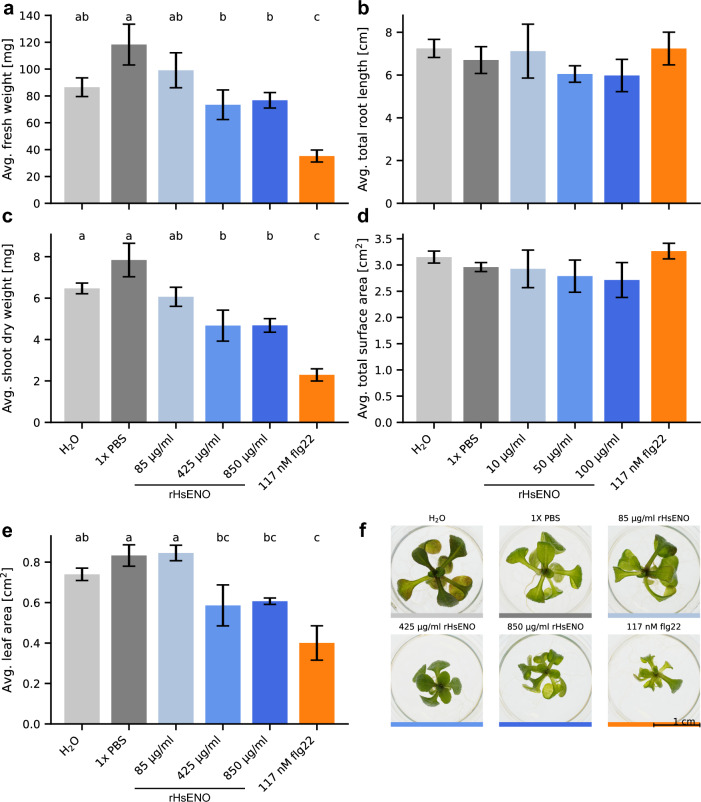
Effect of recombinant *H. schachtii* enolase on growth of *A. thaliana*. Six-day-old *A. thaliana* Col-0 seedlings were grown on MS medium and treated with rHsENO at varying concentrations, water, PBS, or flg22 peptide. **(a, c)** Fresh and dry shoot biomass were significantly reduced by 37.9% and 35.1% following treatment with 50 $\mu$g/ml and 100 $\mu$g/ml rHsENO, respectively, compared to PBS controls, while flg22-treated plants showed stronger reductions (59.3% and 70.2% relative to water control). **(e)** Leaf area decreased by approximately 30% at 425 $\mu$g/ml and 850 $\mu$g/ml rHsENO (29.6% and 27.1%, respectively), whereas 85 $\mu$g/ml had no significant effect. **(f)** Representative plants with leaf areas closest to the mean illustrate the growth-inhibitory effects of high rHsENO concentrations. **(b, d)** Root traits, measured as total root length and total root surface area, showed no significant differences among treatments. Data represent the mean ± SD of n = 9 plants per treatment (except rHsENO 50 $\mu$g/ml in the first repetition, n = 8), averaged across three independent biological replicates. Statistical analysis was performed on biological replicate means.

When pathogens are detected by plants, energy is directed into immune pathways leading to defense activity against pathogens, with the tradeoff of reduced growth and development^46^. To investigate the effect of prolonged exposure of plants to enolase, we grew *A. thaliana* Col-0 in 12-well plates in MS medium solidified with 30% of the standard agar concentration. The 6-day-old seedlings were supplemented with 100 $\mu$l of treatment solution to reach the desired concentration in a final volume of 0.85 ml medium. To test a broad range of concentrations, recombinant enolase was applied at stock concentrations of 85, 425, and 850 $\mu$g/ml, resulting in direct exposure of shoots to these concentrations, whereas dilution in the growth medium yielded final concentrations of 10, 50, and 100 $\mu$g/ml, to which the roots were exposed. For the positive control, plants were treated with 1 $\mu$M flg22, resulting in a final root exposure to 117 nM flg22 peptide. Both the cotyledons and the roots were treated. Plants were phenotyped 14 days after seeding. We assessed leaf area, plant fresh and dry weight, root length and root surface area (Fig. 4) to determine the effect of rHsENO on plant growth and development.

At the lowest tested concentration (85 $\mu$g/ml stock, corresponding to 10 $\mu$g/ml in the medium), rHsENO had no significant effect on leaf area, fresh weight, or shoot dry weight. Only at concentrations of 425 $\mu$g/ml and 850 $\mu$g/ml applied to the shoot, was a reduction in total leaf area of approximately 30% observed (29.63% and 27.14%, respectively; (Fig. 4e).

In comparison, plants treated with flg22 showed a stronger reduction in leaf area of 45.87% relative to the water control and 51.91% relative to PBS. Representative plants with leaf areas closest to the mean illustrate the growth reduction observed at high rHsENO concentrations (Fig. 4f). Fresh and dry shoot biomass were similarly affected. Fresh weight decreased by 37.94% and 35.11% following treatment with 425 $\mu$g/ml and 850 $\mu$g/ml rHsENO, respectively, compared to the PBS control, while flg22 caused reductions of 59.3% and 70.2% relative to water control (Fig. 4a, c). In contrast, no significant differences were detected in root morphology—measured as total root length and total root surface area—between rHsENO-treated and control plants (Fig. 4b, d).

## Discussion

The genome of the cyst nematode *Heterodera schachtii* encodes a single enolase gene, in contrast to humans, yeast, and protozoan parasites, which possess multiple paralogous enolase genes^4–6,47–49^. The presence of a single gene encoding enolase in *H. schachtii* precludes the use of RNAi-mediated knockdown to investigate its function, as this approach cannot distinguish between extracellular and cytosolic expression of the enzyme. The *H. schachtii* enolase is a highly conserved 436 amino acid protein (~47.68 kDa), with all critical active-site residues and Mg$^{2+}$-binding sites conserved except for a single amino acid substitution^38^, compared to yeast enolase. Given that enolase from animal-parasitic nematodes activates immune responses in animal hosts^50,51^, we hypothesized a similar role for plant-parasitic nematode (PPN)-derived enolase, potentially triggering innate immune responses in plants.

We cloned and expressed recombinant enolase from *H. schachtii* in an *E. coli* expression system. Despite utilizing the (DE3)pLysS strain that has been optimized to avoid leaky expression^52^, expression occurred even before IPTG induction and considerable amounts accumulated intracellularly, possibly in inclusion bodies^53^. Nonetheless, sufficient quantities of purified, functionally active protein were successfully obtained. We verified the identity of the purified protein by SDS-PAGE and Western blotting. Furthermore, the recombinant enzyme was able to convert 2-phosphoglycerate to phosphoenolpyruvate, demonstrating its proper folding and function, including the ability to form a homodimer that is necessary for its enzymatic activity^54,55^.

To assess the ability of rHsENO to induce immune responses in plants, we exposed leaf discs of tomato (*Solanum lycopersicum*) and *A. thaliana* to various concentrations of rHsENO. In particular, production of apoplastic reactive oxygen species (ROS) is one of the earliest plant responses to pathogen recognition^40^. Contrary to expectations, we did not observe significant ROS production, indicating no immediate immune response triggered by rHsENO. Prolonged exposure at the highest tested concentrations (425 and 850 $\mu$g/ml) was associated with reduced shoot growth. However, the local concentration of enolase at the plant–nematode interface during infection has not been determined, and quantitative measurements of its abundance during infection are currently lacking. It therefore remains unclear whether the tested concentrations correspond to biologically meaningful exposure levels, and the growth-inhibitory effects observed at high concentrations should be interpreted cautiously. We further note that during the 14-day phenotyping window and the 6-hour qPCR incubation, rHsENO is likely to have undergone some degree of proteolytic turnover in the medium and on the leaf surface. The negative readouts reported here therefore apply to the mixture of intact protein and any degradation products generated under these conditions, rather than to intact rHsENO alone. We cannot distinguish whether the growth inhibition observed at the highest tested concentrations is driven by intact enolase, by degradation-derived peptides, or by non-specific protein overload. Future studies quantifying extracellular enolase concentrations during nematode infection will be necessary to clarify its biological relevance. Concordantly, root treatment with 50 $\mu$g/ml rHsENO did not induce upregulation of canonical immunity markers^41–45^. *FRK1* and *NHL10*, both established positive markers of PTI and HR-associated defense, were instead significantly downregulated. As immune activation is associated with upregulation of these genes, their reduction is inconsistent with classical defense induction. While the absence of ROS production, lack of canonical marker gene induction, and absence of a growth phenotype at the lowest tested concentration are consistent with rHsENO not acting as an elicitor, the assays applied here capture only a subset of plant defense outputs. We cannot exclude that exogenous rHsENO triggers responses not assayed here, such as callose deposition, localized cell wall remodeling, or hormone-independent secondary metabolite induction. The biological significance of the *FRK1* and *NHL10* downregulation, and whether it reflects a specific response to nematode-derived enolase, a generic protein-exposure effect, or a response to enolase as a conserved protein class, cannot be resolved with the present data and represents a target for future investigation using non-enolase protein controls and plant-derived enolase comparisons.

It is important to note that the *E. coli* expression system lacks complex post-translational modifications (PTMs), such as glycosylation or phosphorylation, which could influence enolase function or interactions with the host plant^53,56–58^. *In silico* predictions revealed 30 potential phosphorylation sites (18 Ser, 8 Thr, 4 Tyr) that would be absent in rHsENO, and a single N-glycosylation sequon (N19) with a proline at the X+1 position. Thus, native HsENO is unlikely to carry glycan modifications, while phosphorylation would not be retained in the bacterial expression system. The high structural similarity between nematode and plant enolases could explain the lack of immune recognition by plants through conserved pattern recognition receptors (PRRs)^59,60^, resulting in the plant being unable to identify nematode-derived enolase as non-self. The high similarity could also have a positive effect for nematode parasitism, as it could hamper nematode detection and thereby facilitate parasitism.

In animal systems, enolase engages both the innate and adaptive arms of the immune system. As part of the innate immune response, enolase acts as a pathogen-associated molecular pattern (PAMP) recognized by pattern recognition receptors (PRRs) such as Toll-like receptors (TLRs), including TLR4, often in conjunction with co-receptors like CD14^58,61^. This recognition triggers rapid innate immune signaling cascades, leading to inflammation, recruitment of immune cells, and maturation of antigen-presenting cells (APCs). Through these innate mechanisms, enolase detection initiates the subsequent activation and instruction of the adaptive immune response. Antigen-presenting cells process enolase peptides and present them via major histocompatibility complex (MHC) molecules to T cells, thereby activating both cell-mediated (Th1) and humoral (Th2) responses^16,31,32^. Studies on nematode and fungal enolases illustrate this dual role; enolase triggers immediate innate signaling and also functions as a prominent antigen eliciting specific adaptive immunity in animal hosts. This integration of innate sensing and adaptive antigen presentation underscores the importance of enolase as both an immunostimulant and a target of immune defense in animal-pathogen interactions.

The plasminogen pathway, where $\alpha$-enolase functions as one of multiple receptors for plasminogen^8,62–65^, is completely absent in plants because plants lack the extracellular matrix components such as laminin, fibronectin, and collagen, which are required for plasminogen binding and activation. While animal extracellular matrix is composed of fibrous proteins that serve as substrates for plasmin-mediated degradation and tissue invasion, plant cell walls are instead composed of cellulose, hemicellulose, pectin, and lignin - polysaccharide-based structures that do not interact with the plasminogen system^66^.

Despite detection of extracellular enolase within nematode secretomes^24–27,30,32^, where it often acts as an immunostimulant independently of its conserved glycolytic function, computational analysis (SignalP-6.0) did not predict a canonical secretion signal^39^, leaving the mechanism and biological rationale of its extracellular presence unclear. Enolase is frequently detected in extracellular vesicles^27–29^. Given that the nematode surface coat undergoes continuous renewal, with a turnover rate for *Globodera pallida* estimated between 1 and 2 hours^67^, and that its composition dynamically changes during infection, it is plausible that enolase is released as a byproduct from the secretory vesicles covering the nematode body^68^. This may explain the presence of enolase in nematode secretomes following incubation in water, even though enolase sequences do not code for a signal peptide for secretion. Supporting this, immunolocalization of extracellular vesicle markers actin, GAPDH, and 14-3-3 protein has been demonstrated on the surface of the pine wood nematode *Bursaphelenchus xylophilus*^29,69^. Moreover, cell surface enolase is known to associate with proteins such as heat shock protein 70^70^, another EV marker. This suggests extracellular vesicle-mediated secretion as a significant pathway contributing to the composition of nematode secretomes and source of nematode cell-surface enolase.

It is also important to consider the route of host exposure modeled by our experimental design. Exogenous application of rHsENO to roots approximates surface-coat– or secretome-mediated exposure, consistent with the proposed mechanism of enolase release via extracellular vesicles and continuous surface coat turnover. Whether *H. schachtii* additionally delivers enolase intracellularly via stylet secretion is a distinct question. However, HsENO (Hsc_gene_19655.t1) is not present in the comprehensive effectorome of *H. schachtii* defined by gland cell–specific transcriptomics^71^, arguing against a role as a stylet-delivered effector. Moreover, cyst nematodes migrate predominantly along the apoplastic route prior to syncytium establishment^72^, placing the nematode surface coat and any associated EV-derived proteins in direct contact with the host apoplast during early infection. Our findings should therefore be interpreted as applying specifically to this mode of exposure; an immunomodulatory role for intracellularly delivered enolase, if it occurs, cannot be excluded by the present data.

Together, these results indicate that *H. schachtii* enolase does not function as an elicitor of the canonical plant immune responses tested here. Unlike in animal-parasitic systems, where enolase engages both innate and adaptive immunity, its extracellular role in plant–nematode interactions remains to be defined. Direct experimental evidence demonstrating the localization of enolase at the plant–nematode interface during infection is currently lacking, leaving the functional significance of cell surface-associated enolase in *H. schachtii* an open question.

Several complementary approaches could address these open questions. Transcriptome-wide analysis (RNA-seq) of rHsENO-treated roots would provide an unbiased readout of immune and stress responses beyond the marker-gene panel used here, and could reveal regulatory branches not captured by canonical PTI/HR/JA markers. Direct biochemical assays such as MAPK phosphorylation immunoblots, callose deposition staining, and targeted metabolite profiling for camalexin and indole glucosinolate derivatives would test defense outputs at the protein and metabolite level rather than the transcript level. To address the route-of-exposure limitation, transient expression of HsENO in *Nicotiana benthamiana* via Agrobacterium-mediated infiltration would allow intracellular delivery of the protein and direct comparison with the exogenous application tested here. Immunolocalization using antibodies raised against rHsENO would clarify whether and where enolase accumulates at the plant–nematode interface during *H. schachtii* infection, providing spatial and quantitative context for future functional studies.

## Materials and methods

### Bioinformatic analysis of enolase sequence and protein structure

The coding sequence of *H. schachtii* enolase was obtained from the published transcriptome^35^. Coding sequences of enolase from other organisms were acquired through NCBI (https://www.ncbi.nlm.nih.gov/). The tertiary protein structure was predicted using AlphaFold 3 (https://alphafoldserver.com/^37^). A potential signal peptide for secretion was predicted with SignalP-6.0^39^. The phylogenetic tree for evolutionary analyses was generated in MEGA11^73^ using the Neighbor-Joining method^74^. The bootstrap consensus tree was inferred from 1,000 replicates^75^. Post-translational modifications were predicted using NetPhos 3.1^76^ and NetNGlyc 1.0^77^ with default parameters.

### Isolation of enolase cDNA from *Heterodera schachtii* J2s

Approximately 300 cysts of *H. schachtii* were harvested from a sterile stock culture of mustard (*Sinapis alba cv. Albatros*) and placed in a modified Baermann funnel with 3 mM ZnCl$_2$ to induce hatching. After 5 days, J2s were collected and immediately frozen in liquid nitrogen. RNA was extracted (RNeasy Plant Mini Kit, Qiagen) followed by cDNA synthesis, according to the manufacturer’s instructions (High Capacity cDNA Reverse Transcription Kit, Applied Biosystems, Thermo Fisher). The enolase CDS was amplified from J2 cDNA using primers listed in Supplementary Table S1 with Phusion High-Fidelity DNA Polymerase (Thermo Fisher). The PCR product was separated on a 1% agarose gel. Multiple gel bands were collected and DNA was extracted using QIAquick Gel Extraction Kit (Qiagen), according to the manufacturer’s protocol.

### Cloning, expression and purification of recombinant enolase protein from *H. schachtii*

The purified enolase DNA was ligated into blunt-end vector *pJET1.2/blunt* (Thermo Fisher) and introduced into *E. coli* DH5$\alpha$ Competent Cells (Thermo Fisher) by heat-shock transformation. The sequence was amplified from *pJET1.2/blunt* by PCR using Gateway primers (Primers are listed in Supplementary Table S1) and DNA was purified from agarose gel as described previously. For the BP reaction, the PCR product was ligated into the donor vector *pDONR207* and introduced into the same strain and later into the destination vector (LR reaction) *pDEST17*. The destination vector was transformed into *E. coli* Rosetta (DE3)pLysS Competent Cells (Novagen) for induction of protein expression by addition of 1 mM IPTG (Isopropyl-$\beta$-D-thiogalactopyranoside) at OD$_{600}$ = 0.6. Bacteria were grown in standard LB medium and cells were harvested from a 2 L culture after overnight incubation at room temperature. Bacterial cells were lysed using B-PER Bacterial Protein Extraction Reagent (Thermo Fisher) supplemented with 2 $\mu$l Lysozyme, 2 $\mu$l of DNase I and 1 $\mu$l protease inhibitor cocktail per ml of B-PER reagent, according to the manufacturers instructions. Recombinant enolase was purified by immobilized metal affinity chromatography (IMAC) using Ni-NTA agarose (Qiagen). An imidazole gradient from 50 to 400 mM was used to elute the recombinant protein from the column. Finally, enolase was further purified by dialysis into 1x PBS (137 mM NaCl, 2.7 mM KCl, 10 mM Na$_2$HPO$_4$, 1.8 mM KH$_2$PO$_4$, pH 7.4) or ddH$_2$O with two buffer exchanges (800 ml each) after ~2 hours and again overnight, respectively using $\hbox {Slide-A-Lyzer}^{\textrm{Tm}}$ G3 Dialysis Cassettes (A52976, Thermo Fisher) with a Molecular Weight Cutoff (MWCO) of 20 kDa. Enolase concentration was measured using the Bradford assay according to the manufacturer’s instructions. The purified protein was aliquoted and stored at -80$^{\circ }$C until further use.

### Immunoblotting

Dialyzed recombinant enolase was separated by SDS-PAGE and transferred onto a polyvinylidene fluoride (PVDF) membrane by electroblotting. The membrane was blocked for 1 h at room temperature in TBST (Tris-buffered saline containing 0.1% Tween 20) supplemented with 5% (w/v) non-fat dry milk. The membrane was incubated overnight at 4$^{\circ }$C with gentle agitation in blocking solution containing an anti–polyhistidine-peroxidase antibody (mouse monoclonal, Sigma-Aldrich, A7058) diluted 1:1000. After washing three times for 5 min each in TBST, the membrane was incubated for 1 h at room temperature with an anti-mouse IgG antibody conjugated to horseradish peroxidase (rabbit polyclonal, Sigma-Aldrich, A9044) diluted 1:1000 in blocking solution. The membrane was then washed four times for 5 min each in TBST. Signal detection was performed using a chromogenic substrate solution containing 4-chloro-1-naphthol and hydrogen peroxide. The membrane was incubated in developing solution until bands became visible, rinsed with distilled water to stop the reaction, and documented by scanning. Total protein transfer was subsequently verified by staining the membrane with Ponceau S solution for 20 min. The membrane was destained with distilled water and scanned.

### Enolase activity assay

For the enzymatic activity assay, recombinant enolase was added to 1 ml of 1x PBS supplemented with 10 mM MgSO$_4$ and 2 mM 2-PGA (D-2-Phosphoglyceric acid barium salt hydrate, Sigma-Aldrich, 79480) at pH 7.4 in a semi-micro UV cuvette (10 mm path length, Th. Geyer, 7697105). Phosphoenolpyruvate exhibits a characteristic absorbance peak at 240 nm, whereas 2-phosphoglycerate lacks absorbance at this wavelength^2^. For the positive control, enolase from baker’s yeast (*S. cerevisiae*) (Sigma-Aldrich, E6126) was used; Bovine Serum Albumin (BSA) Fraktion V, NZ-Origin (Carl Roth, Germany, 8076.1) served as the negative control. A concentration of 10 $\mu$g/ml was used for all treatments. Absorbance was monitored at 240 nm at 20 s intervals for 10 minutes using a NanoDrop 2000c spectrophotometer (Thermo Scientific). Measurements were performed using the NanoDrop cuvette position with fixed 1 cm path length. Measurements were repeated in four independent biological replicates.

### Measurement of apoplastic ROS production

*A. thaliana* ecotype *’Columbia’* (Col-0) wild-type seeds maintained as laboratory stock were used in this study. Apoplastic production of reactive oxygen species (H$_{2}$O$_{2}$) was measured in leaf discs of *A. thaliana* Col-0 or tomato (*S. lycopersicum*). Leaf discs of 7-day-old Arabidopsis plants were cut with scissors or with a round cork borer (3 mm diameter). Leaf discs were transferred to a 96-well microplate filled with 200 $\mu$l water and incubated in the dark overnight. The next day, water was removed and replaced with 35 $\mu$l of the luminol derivative L-012 (8-Amino-5-chloro-7-phenylpyrido [3,4-*d*]pyridazine-1,4-(2*H*,3*H*)dione Sodium Salt; Wako, Japan), 15 $\mu$l of 20 $\mu$g/ml horseradish peroxidase and 50 $\mu$l of treatment. Luminescence was measured using a TECAN Infinite 200 Pro microplate reader.

### qRT-PCR analysis

For defense gene analysis, *A. thaliana* Col-0 plants were germinated in 6-well plates containing 1/2-strength MS medium. Seven-day-old seedlings were transferred to fresh plates containing 1 ml 1/2-strength MS medium, with three plants per well. At 12 days post-seeding, plants were treated with recombinant enolase (50 $\mu$g/ml) dialyzed in water or with water as a mock control, in four biological replicates. After 6 h of incubation, root systems were blotted on sterile filter paper, separated, immediately frozen in liquid nitrogen, and stored at -80$^{\circ }$C until further use. Total root RNA was reverse-transcribed into cDNA using the RevertAid First Strand cDNA Synthesis Kit (Thermo Fisher Scientific) per manufacturer’s protocol in 20 $\mu$l reactions. Gene-specific primers (Supplementary Table S2) were designed using NCBI Primer-BLAST with *in silico* specificity screening against the *A. thaliana* genome. Experimental validation was performed via melt curve analysis, which showed single PCR products for all assays. Amplification efficiencies were determined from standard curves generated using 5-fold serial dilutions of pooled cDNA (5-point dilution series, triplicate replicates per dilution point). Efficiency was calculated as $E = 10^{-1/\text {slope}}$ from the linear regression of log$_{10}$(dilution factor) versus Cq. Efficiencies and R$^2$ values for all primer pairs are reported in Supplementary Table S2. qRT-PCR was conducted with Fast SYBR Green Master Mix (Applied Biosystems) on a StepOnePlus Real-Time PCR System (Applied Biosystems). Relative expression was calculated using the Pfaffl method^78^ with measured primer efficiencies. Statistical analysis was performed on $\Delta Ct$ values using Welch’s unpaired t-test with $p < 0.05$ considered statistically significant. Stability of the UBQ10 reference gene was experimentally validated by analyzing Ct values across all samples (control and enolase-treated, n = 4 per group). No significant difference was detected between treatment groups (Welch’s unpaired t-test, p = 0.78), and the standard deviation across all samples was 0.48 cycles, confirming reliable expression under the experimental conditions used in this study.

### Phenotyping of *A. thaliana*

The effect of rHsENO on growth and development was assessed by growing *A. thaliana* (Col-0) in a 12-well plate containing 0.75 ml MS medium solidified with 30% of the standard Daishin agar concentration. Six days after seeding, 100 $\mu$l of water, 1X PBS, recombinant enolase, or flg22 peptide (1 $\mu$M, GenScript) were added directly to each well. Recombinant enolase was applied at stock concentrations of 85, 425, and 850 $\mu$g/ml, resulting in final concentrations of 10, 50, and 100 $\mu$g/ml in the growth medium, respectively. Roots were directly exposed to the final concentrations in the growth medium, while shoots were exposed to the applied stock solutions upon addition to the wells.

Leaf area (EasyLeafArea^79^), fresh weight, and root parameters (EPSON Perfection V700 Photo and WinRHIZO PRO software (http://regent.qc.ca/assets/winrhizo_software.html)) were evaluated 14 days post-seeding. The experiment was repeated in three independent biological replicates. For each independent biological replicate, plant-level measurements (n = 9 plants per treatment per replicate, except rHsENO 50 $\mu$g/ml in the first replicate, n = 8) were averaged to yield one value per treatment per replicate. One-way ANOVA followed by Tukey’s HSD test was then performed on these biological replicate means (n = 3 replicates per treatment) using R, with significance threshold $\alpha$ = 0.05. Exact adjusted p-values for all pairwise comparisons are provided in Supplementary Table S1.

## Supplementary Information

Supplementary Information 1.

Supplementary Information 2.

## Data Availability

All data generated during this study are included in this published article and its Supplementary Information files. This study additionally re-analysed previously published data from Siddique et al., Nature Communications 13, 6190 (2022), https://doi.org/10.1038/s41467-022-33769-w. Specifically, the *H. schachtii* genome assembly and read-coverage data shown in Fig. 1a, and the life-stage-specific RNAseq expression values shown in Fig. 1b (derived from Supplementary Data 4 of that publication), were obtained from this source.
