# Supplementary material for: Heterodera schachtii enolase does not elicit canonical immune responses in Arabidopsis thaliana
Source: Sci Rep. 2026 Jun 8;16:17692. doi: 10.1038/s41598-026-56477-7 (PMC13246771; doi:10.1038/s41598-026-56477-7)
Supplement: Supplementary file 1 — Supplementary Information 1. [file 41598_2026_56477_MOESM1_ESM.docx]

**Supplementary material**

*Heterodera schachtii* enolase does not elicit

canonical immune responses in *Arabidopsis thaliana*

**Maximilian F. Euler^1,*^, Neil Pep Dave N. Sumaya^1,3^, Seema Aslam^1^, Johannes Lerch^1,2^, Badou Mendy^1^ and Florian M.W. Grundler^1^**

^1^Rheinische Friedrich-Wilhelms-Universität Bonn, INRES – Molecular Phytomedicine, Bonn, 53115, Germany

^2^Current address: Rheinische Friedrich-Wilhelms-Universität Bonn, Institut für Molekulare Physiologie und Biotechnologie der Pflanzen (IMBIO), Bonn, 53115, Germany

^3^Current address: Plant Pathology Division, College of Agriculture, University of Southern Mindanao, Kabacan, 9407, Cotabato, Philippines

*max.euler@uni-bonn.de

**Tab. S1** Primer sequences used in this study for cloning of *H. schachtii* enolase.

| **Gene** | **Description** | **F/R** | **Sequence (5'-3')** |
| --- | --- | --- | --- |
| Hsc_gene_19655.t1 | Enolase CDS / Blunt-end cloning | F | ATGTCGGATTCAATCAAACG |
|  |  | R | TTAGTTTTGCGGGTTACGGA |
|  | Enolase Gateway cloning | F | GGGGACAAGTTTGTACAAAAAAGCAGGCTTCATGTCGGATTCAATCAAACG |
|  |  | R | GGGGACCACTTTGTACAAGAAAGCTGGGTATTAGTTTTGCGGGTTACGGA |

**Tab. S2** Primers used for qPCR analysis of Arabidopsis defence genes upon enolase treatment. Primer amplification efficiencies were determined from standard curves (5-point serial dilutions, pooled cDNA template, run in triplicate). Efficiency was calculated as E = 10^(-1/slope) and is reported alongside R² values.

| **Gene** |  | **F/R** | **Sequence (5'-3')** | **Efficiency** | **R^2^** |
| --- | --- | --- | --- | --- | --- |
| AT4G05320 | UBQ10 | F | GGCCTTGTATAATCCCTGATGAATAAG | 1,9091 | 0,9947 |
|  |  | R | AAAGAGATAACAGGAACGGAAACATAGT |  |  |
| AT2G19190 | FRK1 | F | TCGTCGTTCTTTTGACGGCT | 1,9123 | 0,9922 |
|  |  | R | TTCAACGGCCCATTCCTCTC |  |  |
| At5g57220 | CYP81F2 | F | GTCGGCTGCTCACAAAACTC | 2,2278 | 0,9974 |
|  |  | R | TCTCCGTAGTAGCGTCTCCC |  |  |
| AT2G35980 | NHL10 | F | ATTGTCATCCTCGGCGTAGC | 2,0033 | 0,9851 |
|  |  | R | TGTGGTCAAAGCGGGTAAGG |  |  |
| At3g26830 | PAD3 | F | GTGGAGTCGCTGGCATAACA | 2,1627 | 0,9996 |
|  |  | R | GTCCCCAAGTGTTGTCCGAA |  |  |
| At5g13220 | JAZ10 | F | TCGGTAATTCTTCCGACCACTC | 1,9300 | 0,9972 |
|  |  | R | TGATACTAATCTCTCCTTGCGCT |  |  |


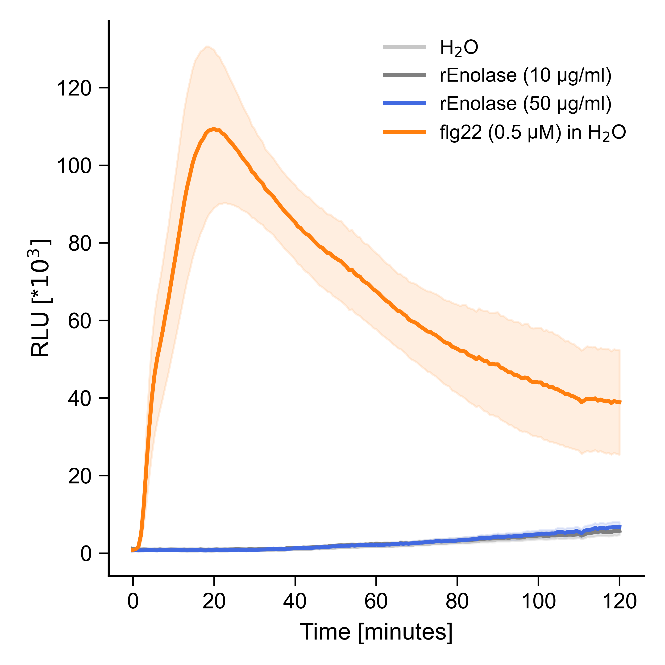

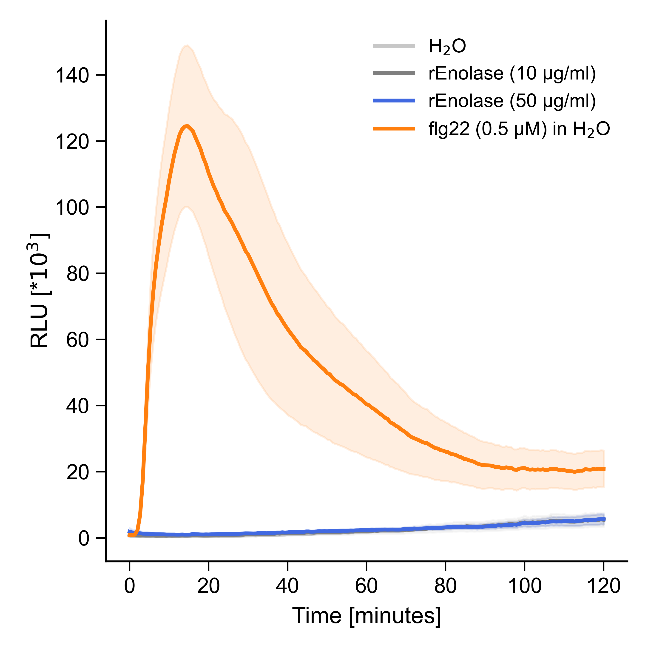


**Fig. S1** ROS measurement in *A. thaliana* leaf discs using 10 µg/ml and 50 µg/ml rHsEno.


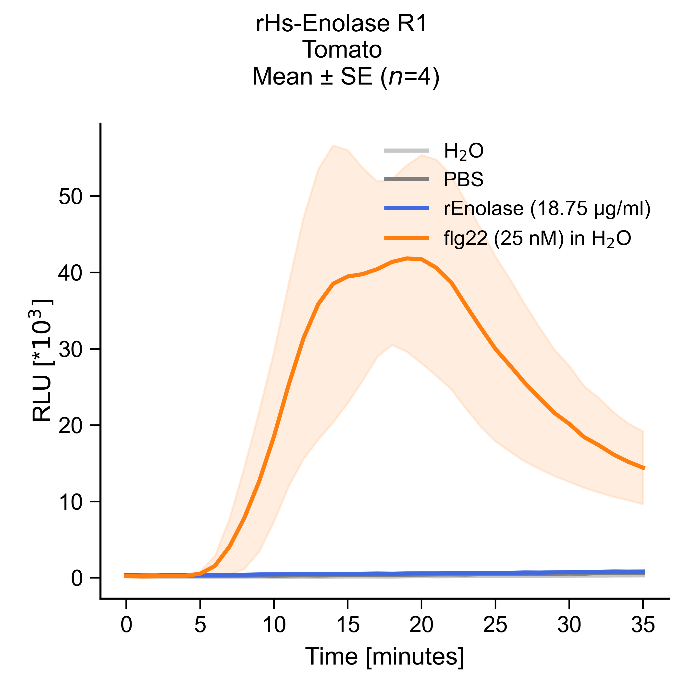

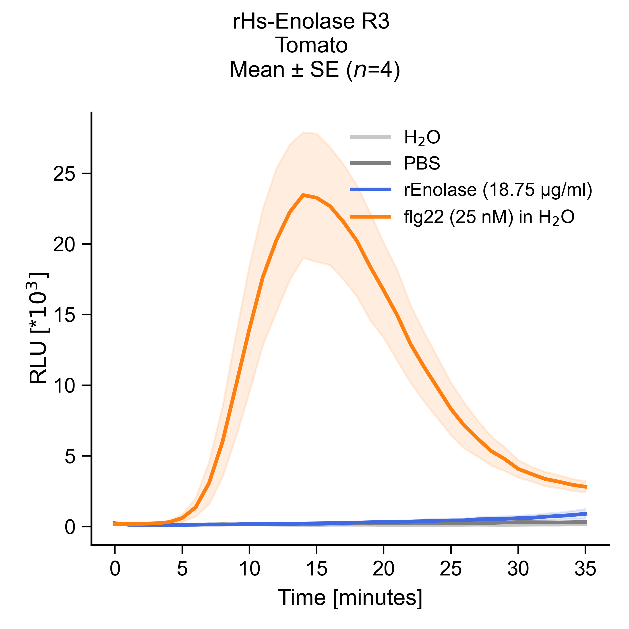


**Fig. S2** ROS measurement in Tomato *(S. lycopersicum)* leaf discs using 18.75 µg/ml rHsEno.


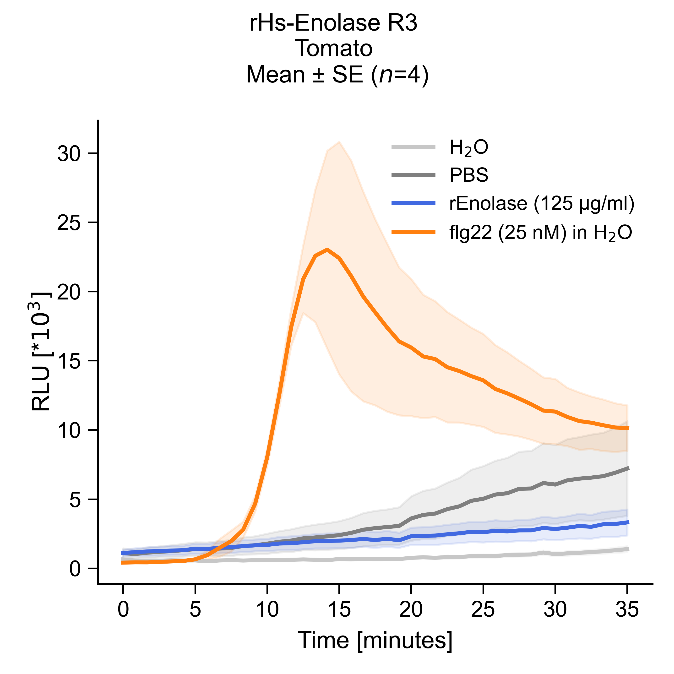

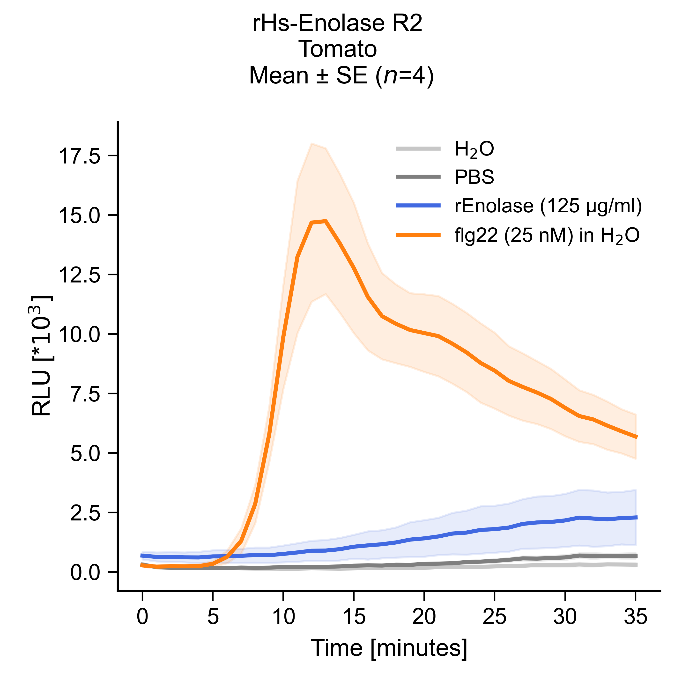


**Fig. S3** ROS measurement in Tomato *(S. lycopersicum)* leaf discs using 125 µg/ml rHsEno.


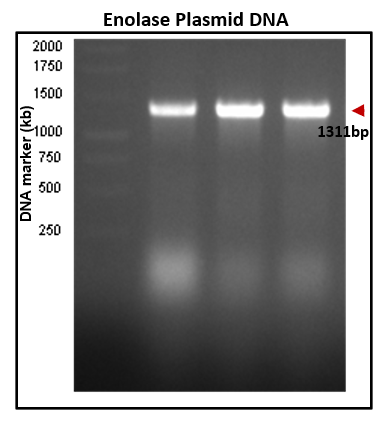


**Fig. S4.** A 1311 bp fraction of enolase was amplified using primers designed for gateway cloning.

**
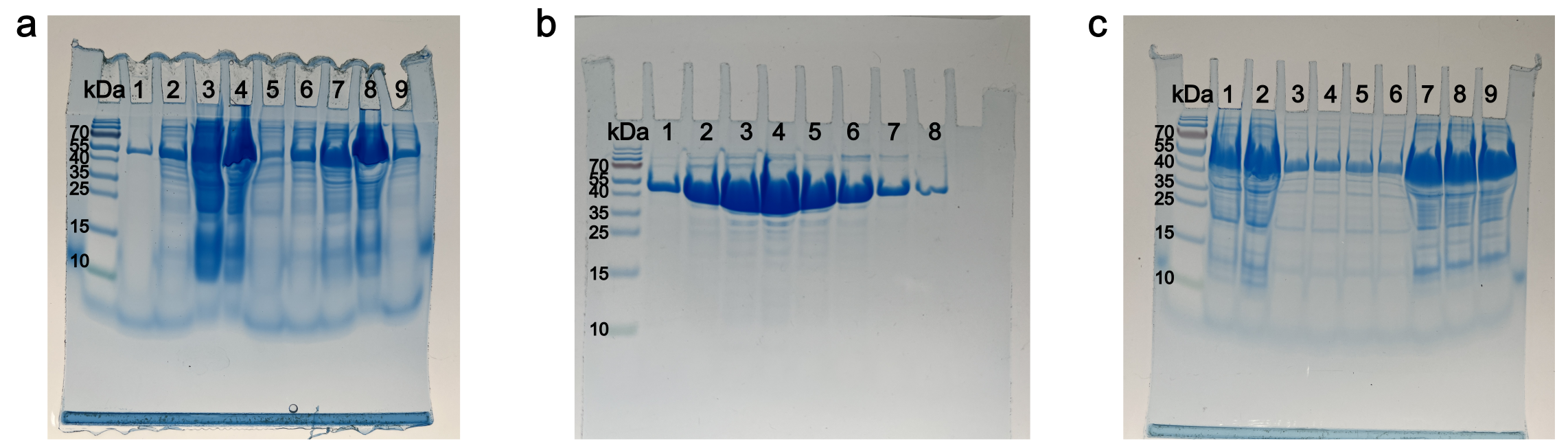
**

**Fig. S5.** Production of recombinant *H. schachtii* enolase. **a)** SDS-PAGE with dialyzed enolase (lane [1]) and analysis of *E. coli* cells before and after induction with 1 mM IPTG, showing uninduced fractions [lanes 2, 6], induced fractions [lanes 3,4, 7,8], and supernatant [lanes 5, 9]. **b)** SDS-PAGE analysis of eluted enolase after IMAC purification. Fraction eluted with 50 mM [1], 100 mM [2], 150 mM [3], 200 mM [4], 250 mM [5], 300 mM [6], 350 mM [7], 400 mM [8], 450 mM [9] imidazole concentration, respectively. **c)** SDS-PAGE analysis of protein samples after extraction of enolase from *E. coli* cells. Bands [1,2, 7-9] represent pellet fraction and soluble lysate [3-6], respectively.


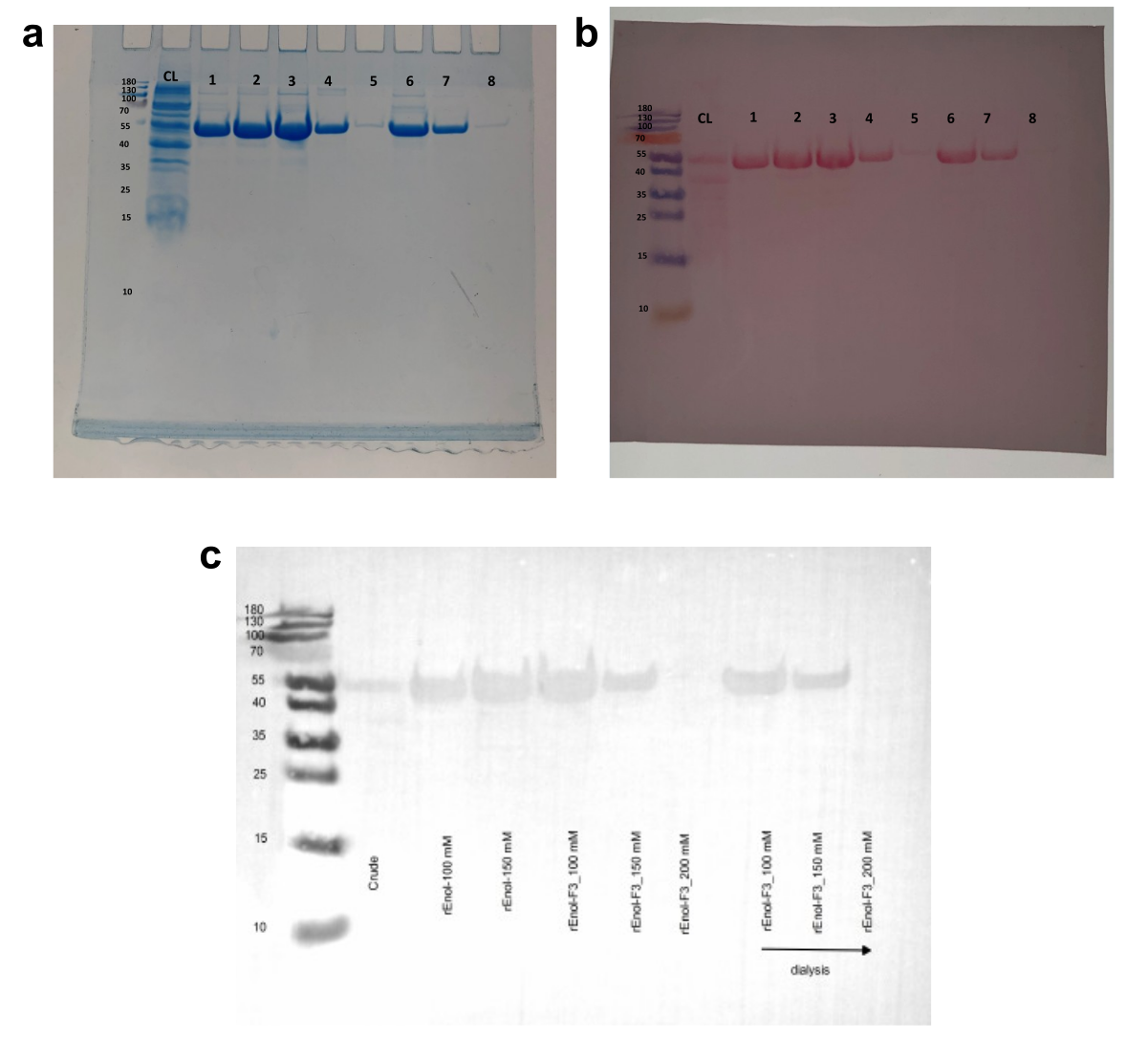


**Fig. S6.** Western blotting with recombinant enolase protein. **a)** SDS-PAGE before transfer on membrane. CL = Crude lysate, enolase eluted with imidazole concentrations of 100, 150, 100, 150 and 100 mM [lanes 1-5, respectively], and dialyzed enolase, eluted fractions with imidazole concentration of 100, 150 and 200 mM [lanes 6-8, respectively]. **b)** Nitrocellulose membrane stained with Ponceau S after detection. **c)** Western blot detection of recombinant enolase using a mouse anti–polyhistidine–peroxidase antibody (1:1000) and anti-mouse IgG–HRP secondary antibody (1:1000). Bands were visualized with 4-chloro-1-naphthol and H₂O₂.
